# Three New Compounds from the Actinomycete *Actinocorallia aurantiaca*

**DOI:** 10.1007/s13659-019-00217-0

**Published:** 2019-09-16

**Authors:** Kai-Yue Han, Xing Wu, Chenglin Jiang, Rong Huang, Zheng-Hui Li, Tao Feng, He-Ping Chen, Ji-Kai Liu

**Affiliations:** 1 School of Pharmaceutical Sciences, South-Central University for Nationalities, Wuhan, 430074 People’s Republic of China; 2 State Key Laboratory for Conservation and Utilization of Bio-Resources in Yunnan, Yunnan Institute of Microbiology, School of Life Sciences, Yunnan University, Kunming, 650091 People’s Republic of China

**Keywords:** Actinomycete, *Actinocorallia aurantiaca*, Polyketides, Anti-NO activity, Antiviral activity

## Abstract

Aurantiadioic acids A (**1**) and B (**2**), two new furan-containing polyketides, and aurantoic acid A (**3**), a new natural product, were isolated from the liquid fermentation of the sika deer dung-derived actinomycete *Actinocorallia aurantiaca*. The structures of the new compounds were established by extensive spectroscopic methods, including 1D & 2D NMR, HRESIMS spectroscopic analysis. The absolute configuration of **3** was assigned by comparison of the specific optical rotations with the reported derivatives. Biological activity evaluations suggested that compounds **1**–**3** showed weak inhibition on NO production in the murine monocytic RAW 264.7 macrophages with IC_50_ values of 35.8, 41.8, 45.2 μM, respectively. Compound **3** showed weak inhibition on influenza A virus (A/PuertoRico/8/1934, H1N1) with an EC_50_ value of 35.9 μM, and a selective index higher than 13.3.

**Electronic supplementary material:**

The online version of this article (10.1007/s13659-019-00217-0) contains supplementary material, which is available to authorized users.

## Introduction

The actinomycetous secondary metabolites, which have attracted great attention from natural product research community in past decades, are considered to be a promising reservoir of new bioactive natural products for drug discovery [1]. The examples of secondary metabolites from actinomycetes, such as streptomycin, actinomycin, tetracycline, rifamycin, vancomycin and mitomycin, etc., have great influence on the treatment of human diseases. The gut microbes from insects have emerged to be fruitful resources for drug leads in recent years [2–4]. However, the gut actinomycetes associated with wild animals have long been underexplored for their potential in drug discovery [5].

In this study, we examined the secondary metabolites of the actinomycete *Actinocorallia aurantiaca* which was isolated from the feces of sika deer. The actinomycete *A. aurantiaca* belongs to the family Thermomonosporaceae, and it has never been chemically investigated. Herein, we report the isolation, structural elucidation, and anti-NO activity of three compounds from the cultures of *A. aurantiaca*.

## Results and Discussion

**Fig. 1:**
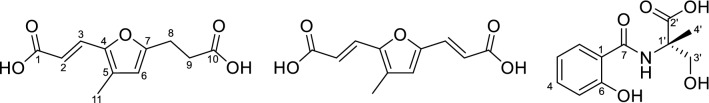
Chemical structures of compounds **1**–**3**

**Fig. 2:**
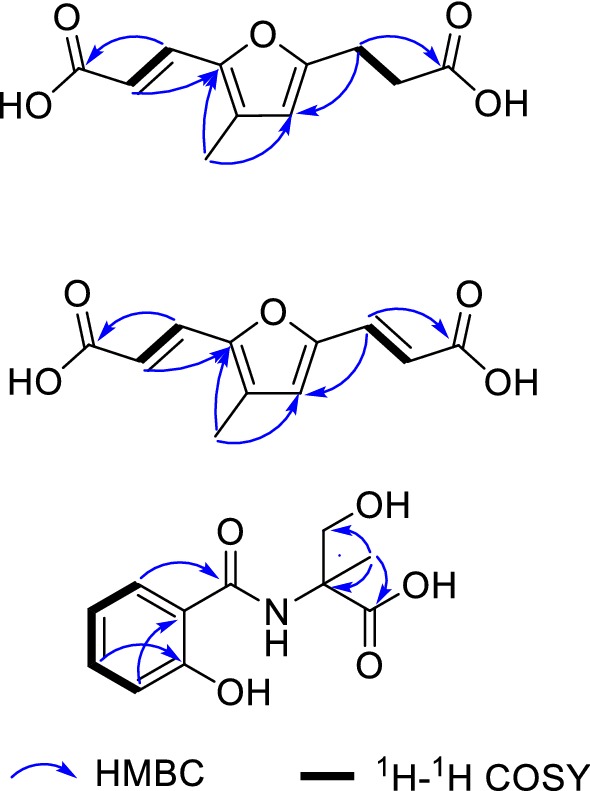
Characteristic mutual HMBC (blue arrow) and ^1^H–^1^H COSY correlations for compounds **1**–**3**

Compound **1** was obtained as a yellow oil. It had a molecular formula of C_11_H_12_O_5_ as determined by (+)-HRESIMS analysis with the protonated ion peak at *m/z* 225.07576 [M+H]^+^ (calcd for C_11_H_13_O_5_, 225.07575), corresponding to six degrees of unsaturation. The ^1^H NMR spectroscopic data of **1** (Table 1) showed the presence of one methyl singlet at *δ*_H_ 2.11 (CH_3_-11), and two *trans*-olefinic methines at *δ*_H_ 6.07 (d, *J* = 15.4 Hz, H-2), 7.44 (d, *J* = 15.4 Hz, H-3), and an olefinic methine singlet at *δ*_H_ 6.12 (s, H-2). The ^13^C NMR and DEPT spectroscopic data (Table 1) presented eleven carbons ascribable to one methyl, two methylenes, three methines, and five quaternary carbons (two carbonyl groups). The spectroscopic features of the chemical shifts at *δ*_C_ 112.2, 129.2, 147.2, and 158.8 implied the presence of a trisubstituted furan ring in compound **1**. Exhaustive analysis of the 2D NMR spectra furnished the establishment of the structure of **1**. The ^1^H–^1^H COSY correlations allowed the connection of C-2–C-3, and C-8–C-9. The HMBC correlations from Me-11 to C-4 (*δ*_C_ 147.2), C-5 (*δ*_C_ 129.2), and C-6 (*δ*_C_ 112.2) indicated the methyl connected to C-5. Furthermore, the *trans*-olefinic protons (H-2, H-3) correlated to a carboxylic group at *δ*_C_ 171.2 (C-1), and C-4 in the HMBC spectrum, indicative of the connection of C-1–C-2–C-3–C-4. Besides, the HMBC correlations from two methylene protons (H-8, H-9) to C-7 and C-10 enabled the connection of C-7–C-8–C-9–C-10 (Fig. 2). Therefore, compound **1** was established to be a furan derivative with two carboxylic groups (Fig. 1), and was given the name aurantiadioic acid A.

**Table 1:** ^1^H NMR and ^13^C NMR spectroscopic data for compounds **1**–**3** (CD_3_OD, *δ* in ppm)

No.	**1**		**2**		**3**	
	*δ*_C_	*δ*_H_	*δ*_C_	*δ*_H_	*δ*_C_	*δ*_H_
1	171.2, C		170.8, C		118.3, C	
2	113.4, CH	6.07, d (15.4)	117.8, CH	6.32, d (15.6)	130.1, CH	7.83, d (8.0)
3	130.7, CH	7.44, d (15.4)	129.5, CH	7.47, d (15.6)	120.4, CH	6.91, t (4.4)
4	147.2, C		150.2, C		134.8, CH	7.36, t (7.7)
5	129.2, C		129.4, C		118.3, CH	6.90, d (5.7)
6	112.2, CH	6.12, s	120.2, CH	6.72, s	160.4, C	
7	158.8, C		153.2, C		169.6, C	
8	24.7, CH_2_	2.94, t (7.3)	131.5, CH	7.36, d (15.8)		
9	33.0, CH_2_	2.65, t (7.3)	119.7, CH	6.39, d (15.8)		
10	176.1, C		170.5, C			
11	10.3, CH_3_	2.11, s	10.1, CH_3_	2.17, s		
1′					62.9, C	
2′					177.0, C	
3′					66.1, CH_2_	3.96, d (11.0) 4.03, d (11.0)
4′					20.6, CH_3_	1.60, s

^1^H NMR were measured at 600 MHz; ^13^C NMR were measured at 150 MHz

Compound **2** was obtained as a yellow oil. The molecular formula of C_11_H_10_O_5_ was determined by the (+)-HRESIMS protonated ion peak at *m/z* 223.06007 [M+H]^+^, and sodium-adduct ion peak at *m/z* 245.04192 [M+Na]^+^, corresponding to seven degrees of unsaturation. The ^1^H and ^13^C NMR spectroscopic data of **2** (Table 1) showed highly similarities to those of **1**, indicating that it was a congener of **1**. The presence of an additional carbon double bond at *δ*_C_ 119.7 (C-9), 131.5 (C-8) of **2** compared to those of **1** was assigned by the HMBC correlations from the *trans*-double bond protons H-8 and H-9 to C-7 (*δ*_C_ 153.2) and C-10 (*δ*_C_ 170.5) (Fig. 2). Thus, the structure of **2** was established as shown in Fig. 1, and was trivially named as aurantiadioic acid B.

The yellow oil compound **3**, possessed the molecular formula of C_11_H_13_O_5_N as determined by the (+)-HRESIMS sodium-adduct ion peak at *m/z* 262.06842 [M+Na]^+^ (calcd for C_11_H_13_O_5_NNa, 262.06914), indicating six indices of hydrogen deficiency. The ^1^H NMR spectroscopic data of **3** (Table 1) showed the presence of an *ortho*-substituted benzene ring at *δ*_H_ 7.83 (*J* = 8.0 Hz, H-2), 6.90 (*J* = 5.7 Hz, H-5), *δ*_H_ 6.91(*J* = 4.4 Hz, H-3), and 7.36 (*J* = 7.7 Hz, H-4), which also confirmed by the ^1^H–^1^H COSY correlations of H-2/H-3/H-4/H-5. The HMBC correlations from H-2 to a carbonyl at *δ*_C_ 169.6 (C-7) indicated the attachment of a carbonyl at C-1. The down-field chemical shifts of C-6 (*δ*_C_ 160.4) suggested the presence of a hydroxy substituent. Furthermore, the methyl singlet at *δ*_H_ 1.60 showed HMBC correlations to the hydroxymethyl at *δ*_C_ 66.1 (C-3′), the quaternary carbon at *δ*_C_ 62.9 (C-1′), and the carboxylic group at *δ*_C_ 177.0 (C-2′) suggested the presence of an isolated unit assembled by C-1′ to C-4′. The nitrogen atom was assigned between C-7 and C-1′ based on the chemical shifts of C-7 and C-1′ to satisfy the element composition of the molecular formula. Thus, compound **3** was established as shown in Fig. 1. However, although this compound was recorded in SciFinder database, but there was no literature information available. We herein reported the chemical shifts, and first origin organism of this compound.

**Fig. 3:**
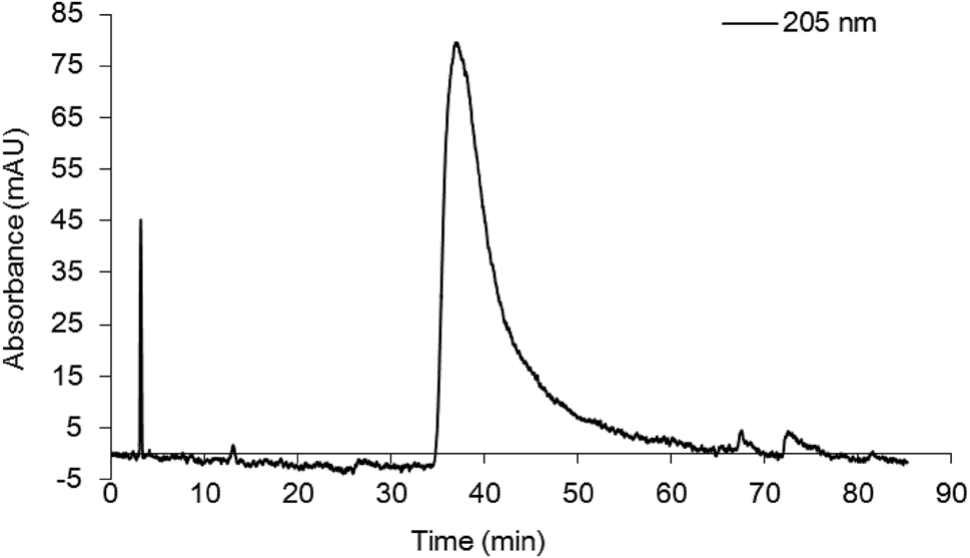
Chiral-phase HPLC analysis of **3**

When examining the structure of **3**, it was possibly generated by dehydration of an anthranilic acid and an unusual amino acid 2-methylserine. Since compound **3** harbored a sole chiral center, it was subjected to the chiral-phase HPLC analysis to investigate the optical purity. As depicted in Fig. 3, the analysis result suggested that it presented in enantiomerically pure form. Thus, the absolute configuration of **3** was assigned as 1′*S* according to the specific optical rotatory data ([*α*] + 17.2) compared with the reported specific optical rotatory values for (−)-2-methyl-d-serine (2*R*, [*α*] − 6.0) and (+)-2-methyl-l-serine (2*S*, [*α*] + 6.0) [6].

Compounds **1**–**3** were evaluated for their inhibition against NO production in murine monocytic RAW 264.7 macrophages. As a result, compounds **1**–**3** displayed weak inhibitory activity with IC_50_ values of 35.8, 41.8, 45.2 μM, respectively. The IC_50_ value for the positive control PDTC (ammonium pyrrolidine dithiocarbamate) was 15.3 μM.

Compound **3** was further screened for inhibitory activity against the influenza virus strain A/PuertoRico/8/1934 (H1N1). The result suggested that **3** displayed weak inhibition on the virus A/PuertoRico/8/1934 with an EC_50_ value of 35.9 μM, and a selective index higher than 13.3.

## Experimental

### General Experimental Procedures

Optical rotations were obtained on an Autopol IV Automatic Polarimeter (Rudolph Research Analytical, Hackettstown, NJ, USA). UV spectra were recorded on a Hitachi UH5300 UV–Vis spectrophotometer (Hitachi, Ltd., Tokyo, Japan). An IRTracer-100 Fourier transform infrared spectrophotometer (Shimazu Corporation, Kyoto, Japan) was used for scanning IR spectroscopy using KBr pellets. 1D and 2D NMR spectra were obtained on Bruker Ascend 600 MHz spectrometers (Bruker Corporation, Karlsruhe, Germany). HRESIMS were recorded on a Q Exactive HF Mass Spectrometer (Thermo Fisher Scientific, Waltham, MA, USA). Sephadex LH-20 (Amersham Biosciences, Uppsala, Sweden) and silica gel (Qingdao Haiyang Chemical Co., Ltd., Qingdao, China) were used for column chromatography (CC). Medium pressure liquid chromatography (MPLC) was performed on a Büchi Sepacore System equipped with pump manager C-615, pump modules C-605 and fraction collector C-660 (Büchi Labortechnik AG, Flawil, Switzerland), and columns packed with Chromatorex C-18 (dimensions 450 mm × i.d. 14 mm, particle size: 40–75 μm, Fuji Silysia Chemical Ltd., Kasugai, Japan). Chiral-phase HPLC analysis were conducted on an Agilent 1260 Infinity II liquid chromatography system with a Diacel Chiralpak AD-H column (i.d. 4.6 mm × 250 mm, 1 mL min^−1^), eluting with *n*-hexane–isopropanol 90:10. Preparative high performance liquid chromatography (prep-HPLC) were performed on an Agilent 1260 Infinity II liquid chromatography system equipped with a Zorbax SB-C18 column (particle size 5 μm, dimension 150 mm × i.d. 9.4 mm, flow rate 5 mL min^−1^, respectively) and a DAD detector (Agilent Technologies, Santa Clara, CA, US).

### Actinomycete Material

The strain was identified as *Actinocorallia aurantiaca* by Prof. Shen Qin of Yunnan University. A voucher strain (YIM 111109) was deposited at the School of Pharmaceutical Sciences, South-Central University for Nationalities, China.

The actinomycete *A. aurantiaca* strain was cultured in 50 500-mL Erlenmeyer flasks with the liquid culture medium consist of glucose 20 g, peptone 2 g, yeast extract 2 g, soluble starch 5 g, K_2_HPO_4_ 0.5 g, MgSO_4_ 0.5 g, NaCl 4 g, CaCO_3_ 2 g in 1 L of deionized water, the pH was adjusted to 7.8 before autoclaving. All flasks were incubated at 25 °C and shaking at 150 rpm for 25 days.

### Extraction and Isolation

The total liquid culture (20 L) was evaporated to 5 L, then extracted with EtOAc for four times to obtain a total extract 19.2 g. The crude extract was eluted on MPLC with a stepwise gradient of MeOH/H_2_O (0–100%) to afford eight fractions (A–H).

Fraction H (3.2 g) was applied to silica gel column chromatography eluting with petroleum ether/acetone (5:1–2:1) to give ten subfractions (H1–H10). Subfraction H3 was purified by prep-HPLC (MeCN/H_2_O = 3:97 → 23:77, 5 mL min^−1^, 25 min) to yield compounds **2** (3.1 mg, t_*R*_ = 15.1 min) and **3** (8.2 mg, t_*R*_ = 20.3 min). Fraction G (2.1 g) was subjected to Sephadex LH-20 (acetone) to furnish five subfractions (G1–G3). Subfraction G2 was purified on prep-HPLC (MeCN/H_2_O = 15:85, isocratic, 5 mL min^−1^, 25 min) to yield compound **1** (10.3 mg, t_*R*_ = 12.5 min).

### Spectroscopic Data Of Compounds

#### Aurantiadioic Acid A (**1**)

Yellow oil; UV (MeOH) λ_max_ (log *ε*) 215.0 (2.66); IR (KBr) *ν*_max_ 3338, 2943, 2381, 1454, 1114, 1031 cm^−1^; ^1^H NMR (600 MHz, CD_3_OD) and ^13^C NMR (150 MHz, CD_3_OD) data: Table 1; HRESIMS *m/z* 225.07576 [M + H]^+^ (calcd for C_11_H_13_O_5_, 225.07575).

#### Aurantiadioic Acid B (**2**)

Yellow oil; UV (MeOH) λ_max_ (log *ε*) 325.0 (4.57); IR (KBr) *ν*_max_ 3338, 2943, 2831, 1452, 1114, 1031 cm^−1^; ^1^H NMR (600 MHz, CD_3_OD) and ^13^C NMR (150 MHz, CD_3_OD) data: Table 1; HRESIMS *m/z* 223.06007 [M+H]^+^, 245.04192 [M+Na]^+^ (calcd for C_11_H_11_O_5_, 223.06065, C_11_H_10_O_5_Na, 245.04259).

#### Aurantoic Acid A (**3**)

Yellow oil; [*α*] − 16.9 (*c* 0.2, MeOH); UV (MeOH) λ_max_ (log *ε*) 235.0 (3.24); IR(KBr) *ν*_max_ 3342, 2943, 2831, 1452, 1148, 1031; ^1^H NMR (600 MHz, CD_3_OD) and ^13^C NMR (150 MHz, CD_3_OD) data: Table 1; HRESIMS *m/z* 262.06842 [M+Na]^+^ (calcd for C_11_H_13_O_5_NNa, 262.06914).

### Nitric Oxide Inhibitory Assay

The procedures of nitric oxide inhibitory assay were similar with those in previously reported literature [7]. Moreover, PDTC (ammonium pyrrolidine dithiocarbamate) was used as positive control in this research.

### Viral Replication Inhibition Assay

The viral replication inhibition assay against the influenza virus strain A/PuertoRico/8/1934 (H1N1) was performed with the procedures that similar with those reported in the literature [8].

## Electronic supplementary material

Below is the link to the electronic supplementary material.

Supplementary file1 (DOCX 1207 kb)
