# Supplementary material for: Three New Compounds from the Actinomycete Actinocorallia aurantiaca
Source: Nat Prod Bioprospect. 2019 Sep 16;9(5):351–4. doi: 10.1007/s13659-019-00217-0 (PMC6814668; doi:10.1007/s13659-019-00217-0)
Supplement: Supplementary file 1 — Supplementary file1 (DOCX 1207 kb) [file 13659_2019_217_MOESM1_ESM.docx]

**Supplementary data for**

**Three New Compounds from the Actinomycete *Actinocorallia aurantiaca***

Kai-Yue Han,^a^ Xing Wu,^a^ Chenglin Jiang,^b^ Rong Huang,^a^ Zheng-Hui Li,^a^ Tao Feng,^a^ He-Ping Chen,^a,^* Ji-Kai Liu^a,^*

^a^School of Pharmaceutical Sciences, South-Central University for Nationalities, Wuhan 430074, China

^b^State Key Laboratory for Conservation and Utilization of Bio-Resources in Yunnan, Yunnan Institute of Microbiology, School of Life Sciences, Yunnan University, Kunming 650091, People’s Republic of China

Corresponding authors:

E-mails: chenhp@mail.scuec.edu.cn (H.-P. Chen); jkliu@mail.kib.ac.cn (J.-K. Liu)

**Contents**

[Figure S1. ^1^H NMR spectrum of **1** (600 MHz, CD_3_OD). S2](#_Toc15830023)

[Figure S2. ^13^C NMR and DEPT spectra of **1** (150 MHz, CD_3_OD). S3](#_Toc15830024)

[Figure S3. HSQC spectrum of **1**. S4](#_Toc15830025)

[Figure S4. ^1^H-^1^H COSY spectrum of **1**. S5](#_Toc15830026)

[Figure S5. HMBC spectrum of **1**. S6](#_Toc15830027)

[Figure S6. HREIMS report of **1**. S7](#_Toc15830028)

[Figure S7. ^1^H NMR spectrum of **2** (600 MHz, CD_3_OD). S8](#_Toc15830029)

[Figure S8. ^13^C NMR and DEPT spectra of **2** (150 MHz, CD_3_OD). S9](#_Toc15830030)

[Figure S9. HSQC spectrum of **2**. S10](#_Toc15830031)

[Figure S10. ^1^H-^1^H COSY spectrum of **2**. S11](#_Toc15830032)

[Figure S11. HSBC spectrum of **2**. S12](#_Toc15830033)

[Figure S12. HREIMS report of **2**. S13](#_Toc15830034)

[Figure S13. ^1^H NMR spectrum of **3**(600 MHz, CD_3_OD). S14](#_Toc15830035)

[Figure S14. ^13^C NMR and DEPT spectra of **3** (150 MHz, CD_3_OD). S15](#_Toc15830036)

[Figure S15. HSQC spectrum of **3**. S16](#_Toc15830037)

[Figure S16. ^1^H-^1^H COSY spectrum of **3**. S17](#_Toc15830038)

[Figure S17. HMBC spectrum of **3**. S18](#_Toc15830039)

[Figure S18. HREIMS report of **3**. S19](#_Toc15830040)

[Figure S19. Chiral-phase HPLC analysis of **3**. S20](#_Toc15830041)

# Figure S1. ^1^H NMR spectrum of 1 (600 MHz, CD_3_OD).


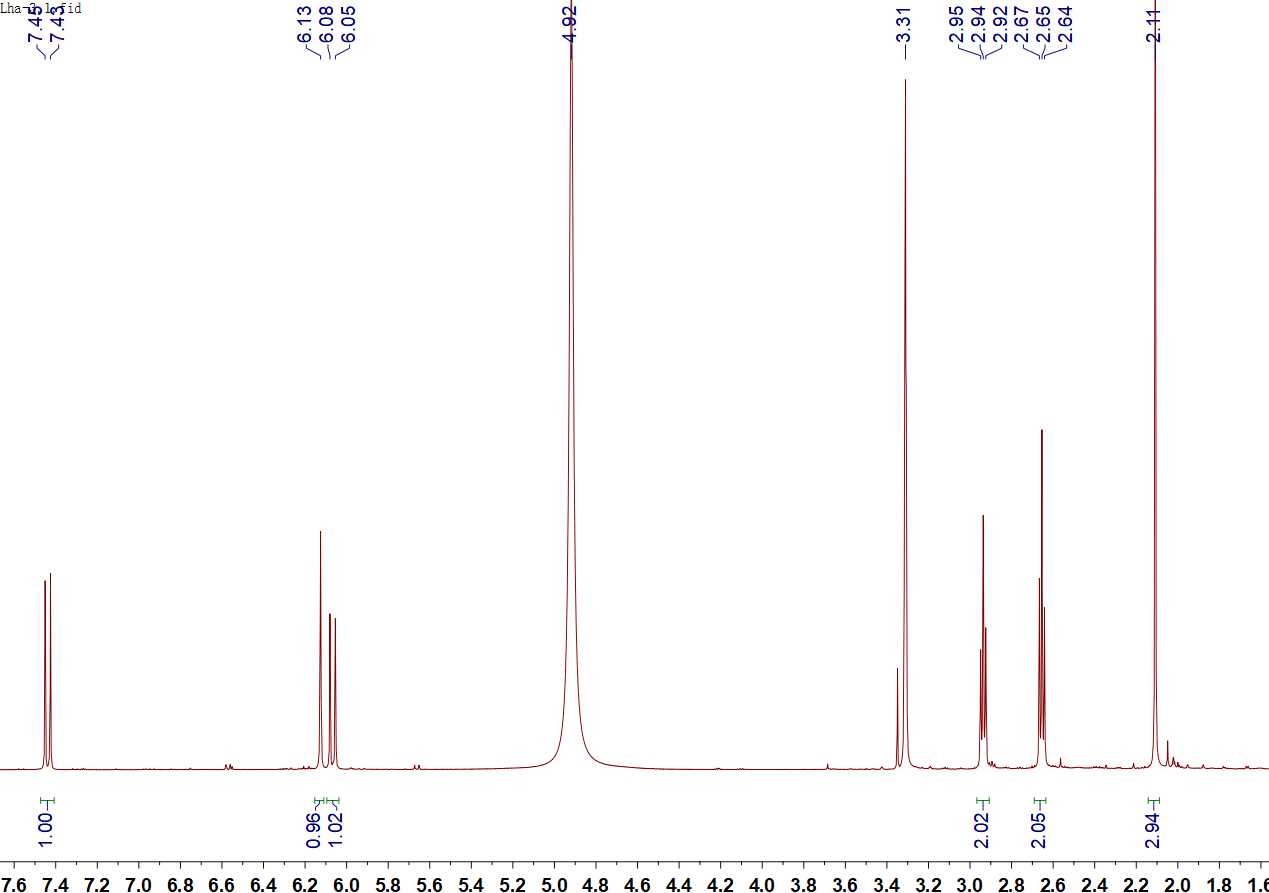


# Figure S2. ^13^C NMR and DEPT spectra of 1 (150 MHz, CD_3_OD).


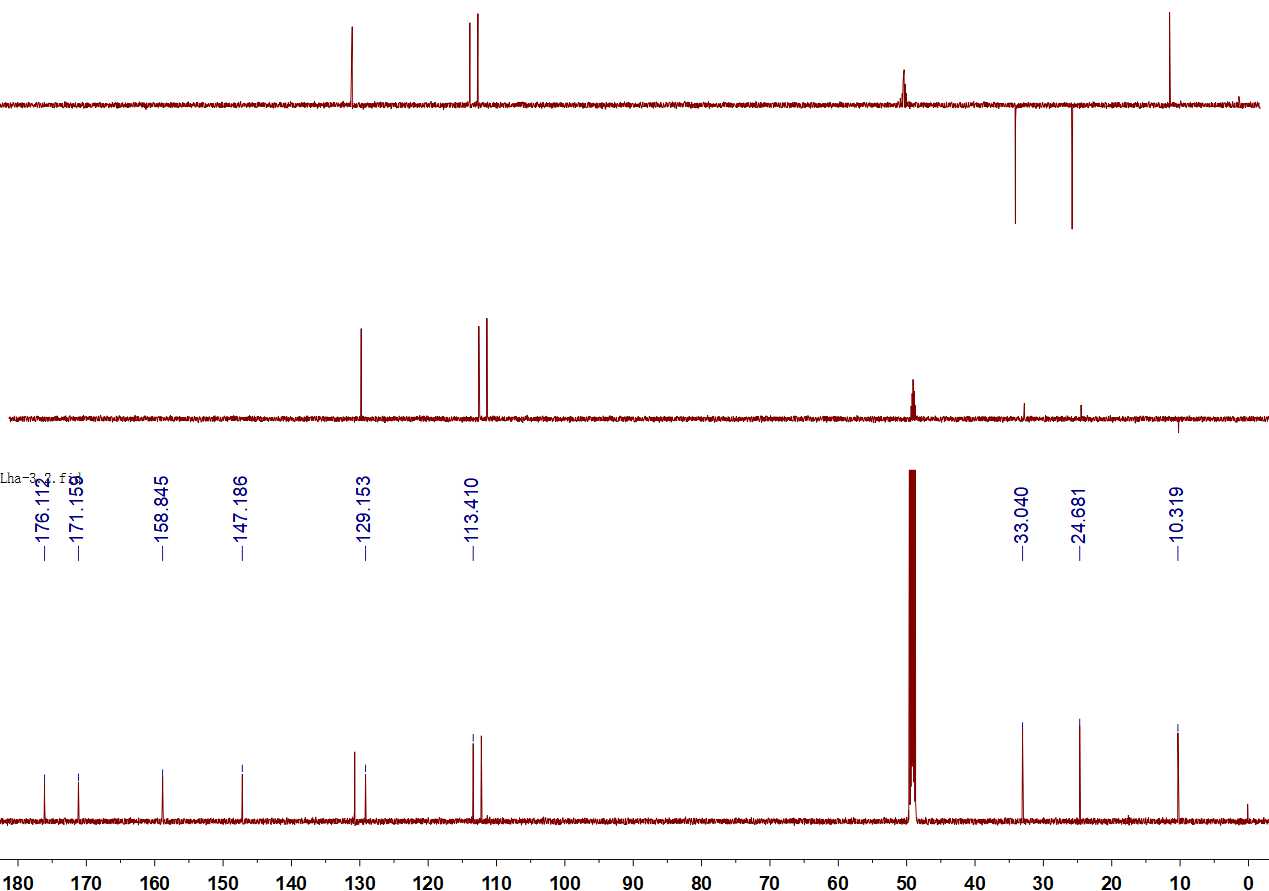


# Figure S3. HSQC spectrum of 1.


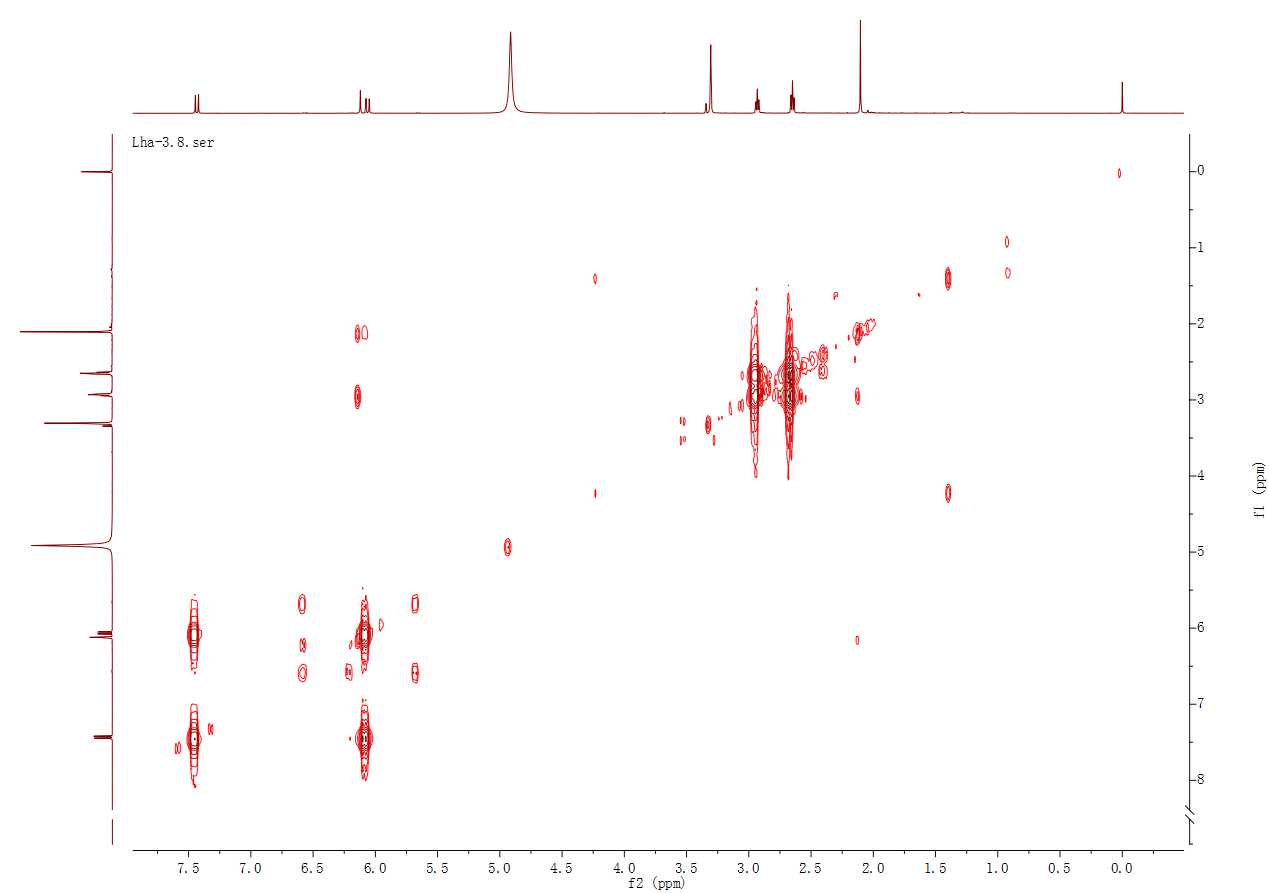


# Figure S4. ^1^H-^1^H COSY spectrum of 1.


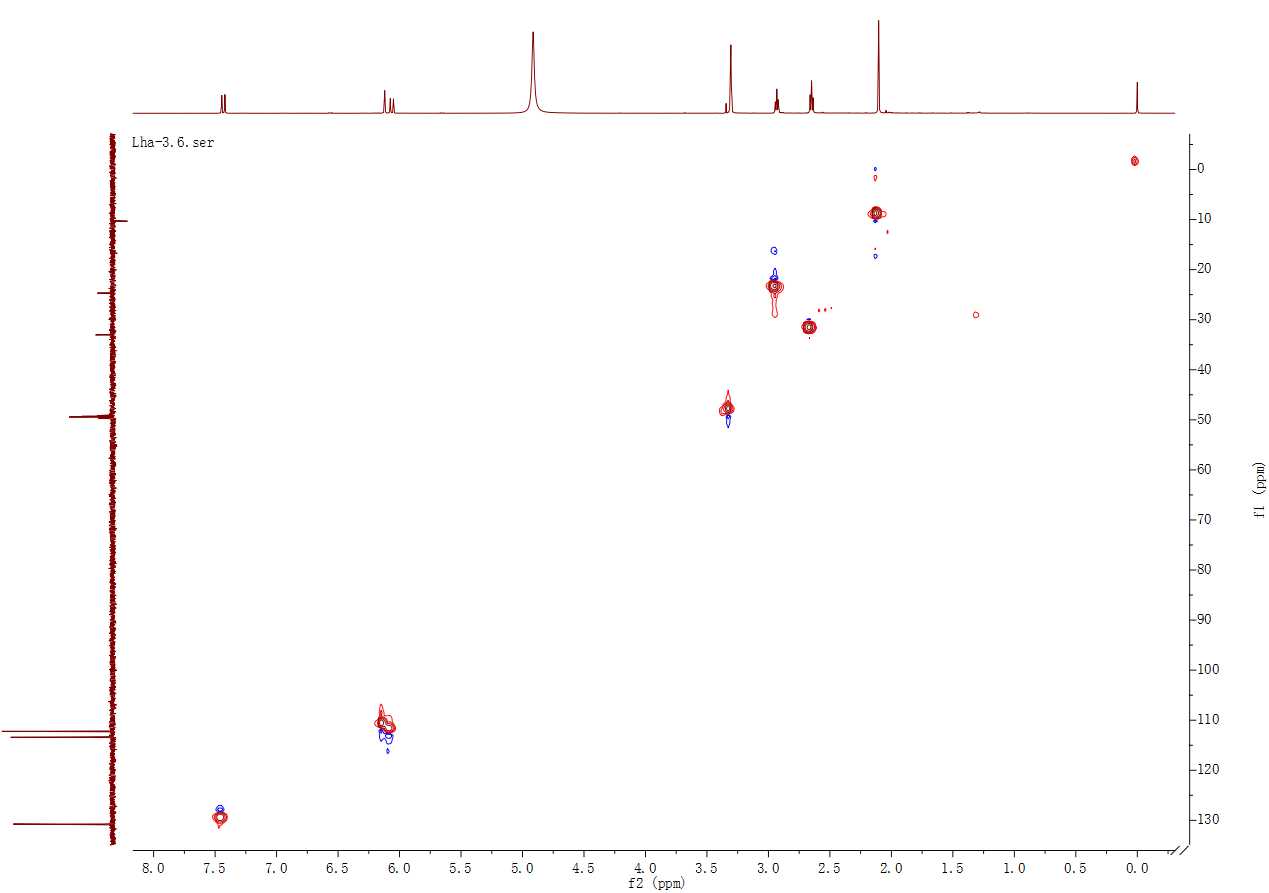


# Figure S5. HMBC spectrum of 1.


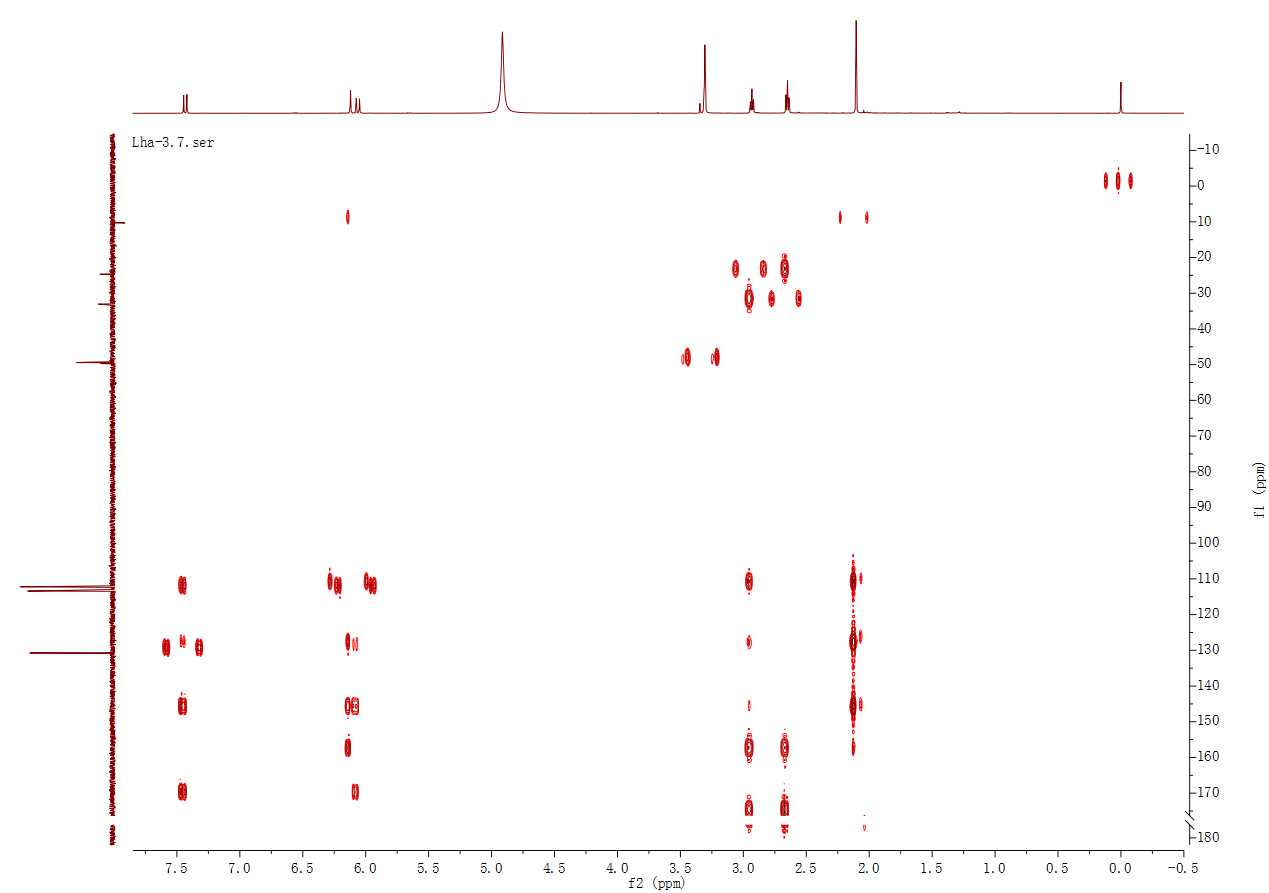


# Figure S6. HREIMS report of 1.


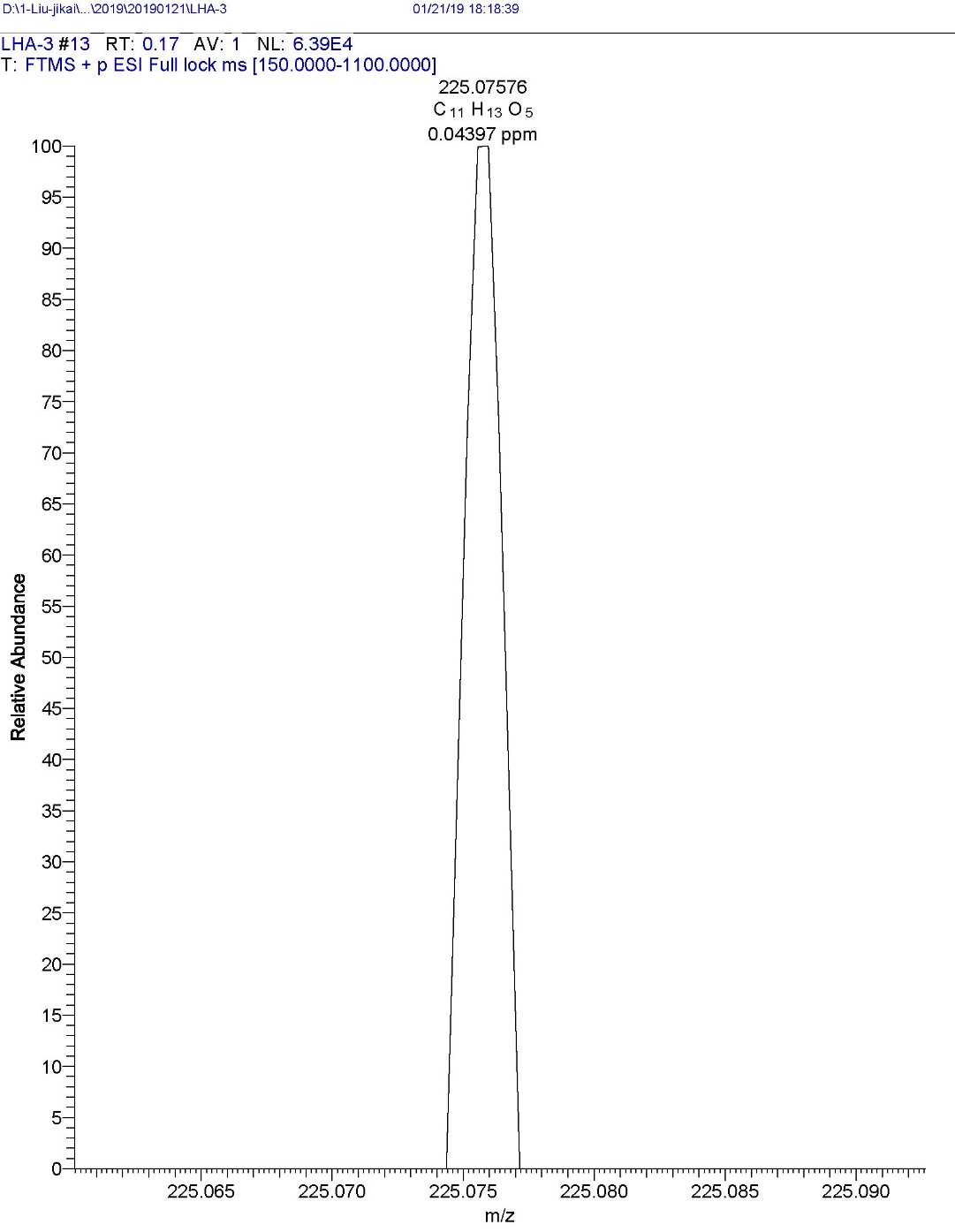


# Figure S7. ^1^H NMR spectrum of 2 (600 MHz, CD_3_OD).


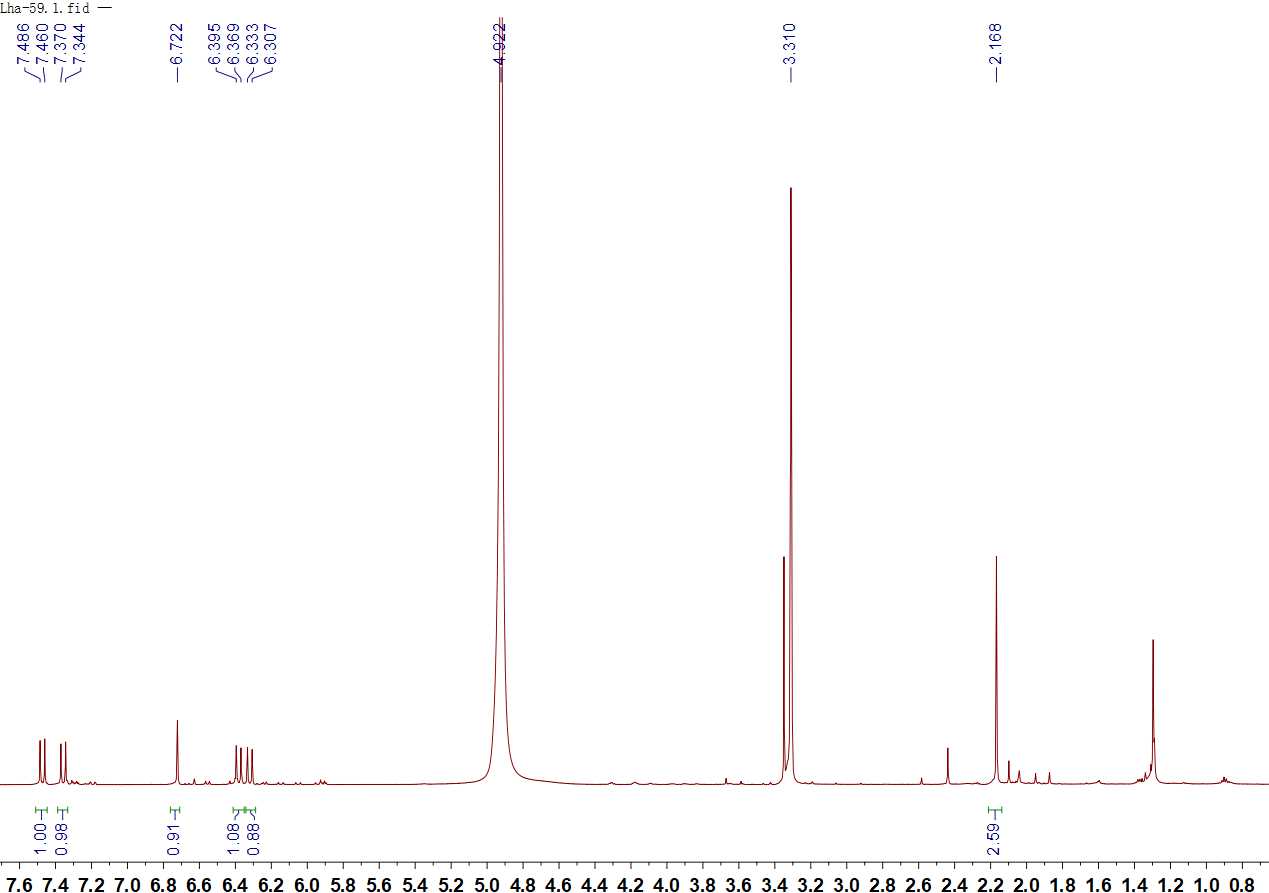


# Figure S8. ^13^C NMR and DEPT spectra of 2 (150 MHz, CD_3_OD).


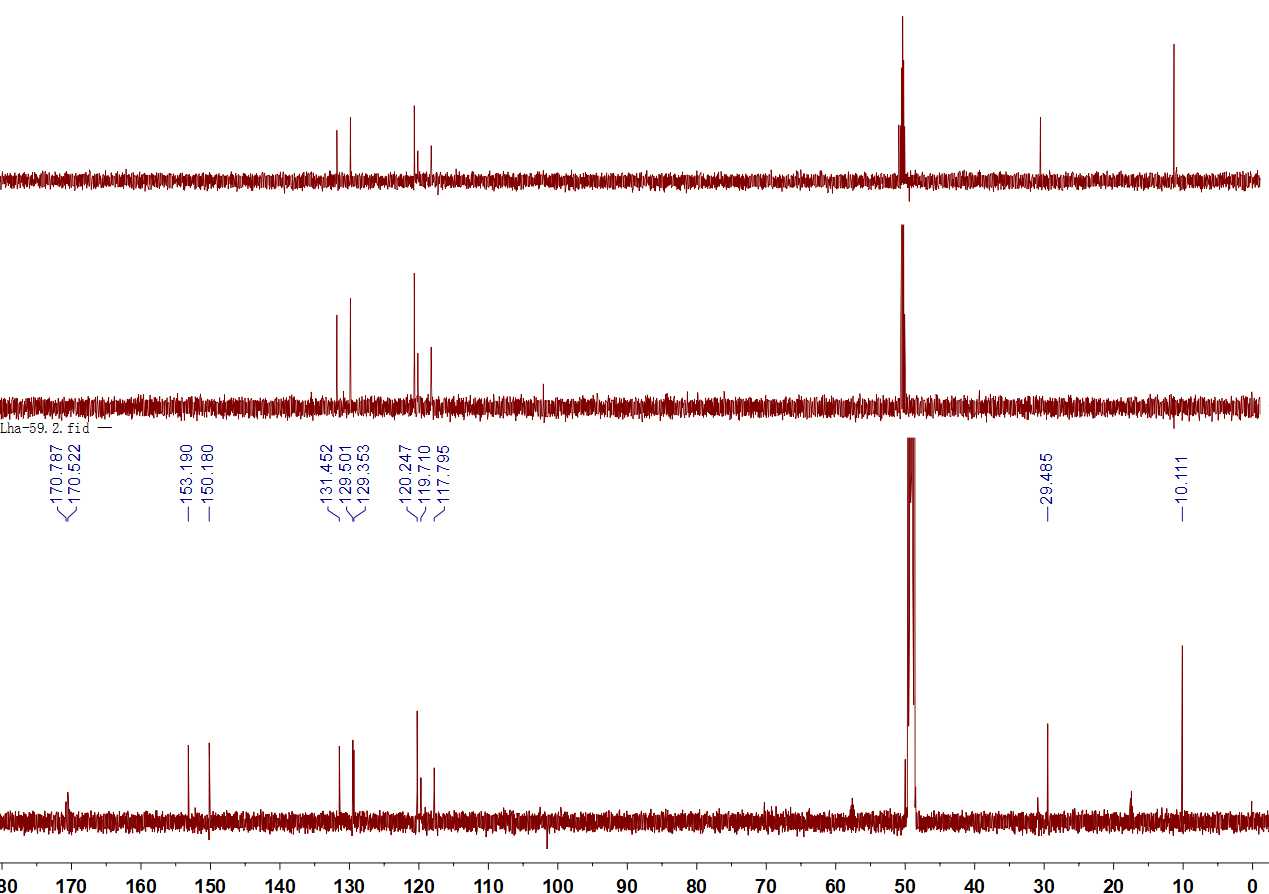


# Figure S9. HSQC spectrum of 2.


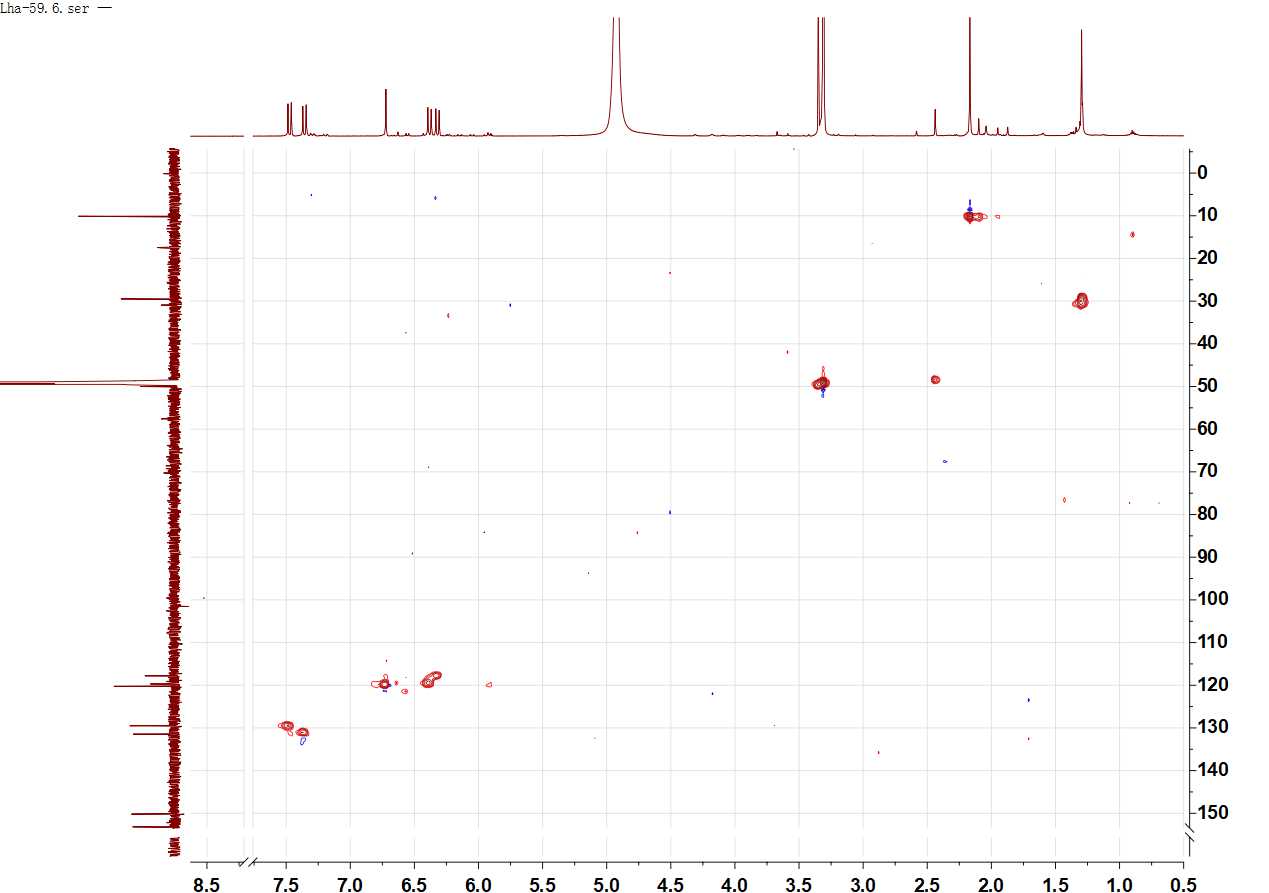


# Figure S10. ^1^H-^1^H COSY spectrum of 2.


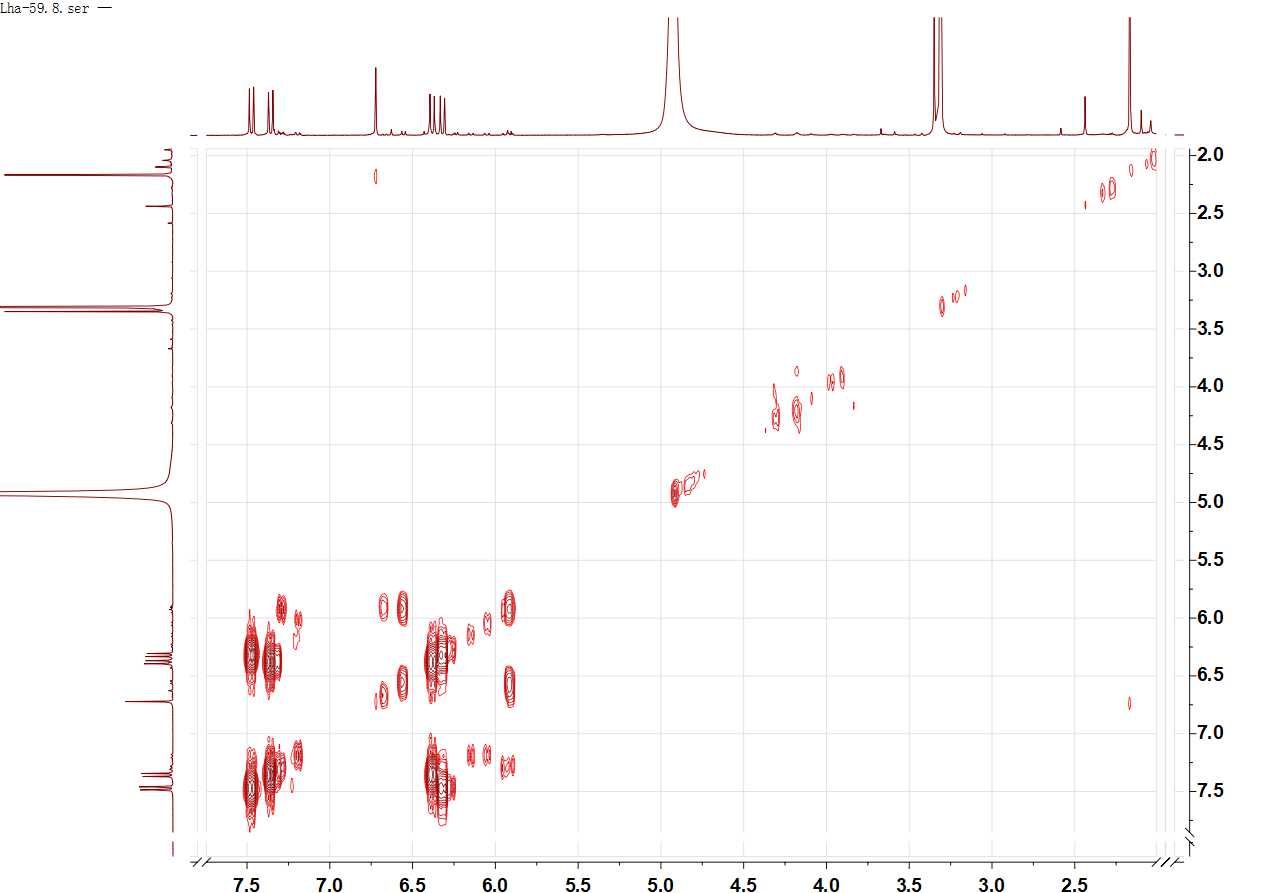


# Figure S11. HSBC spectrum of 2.


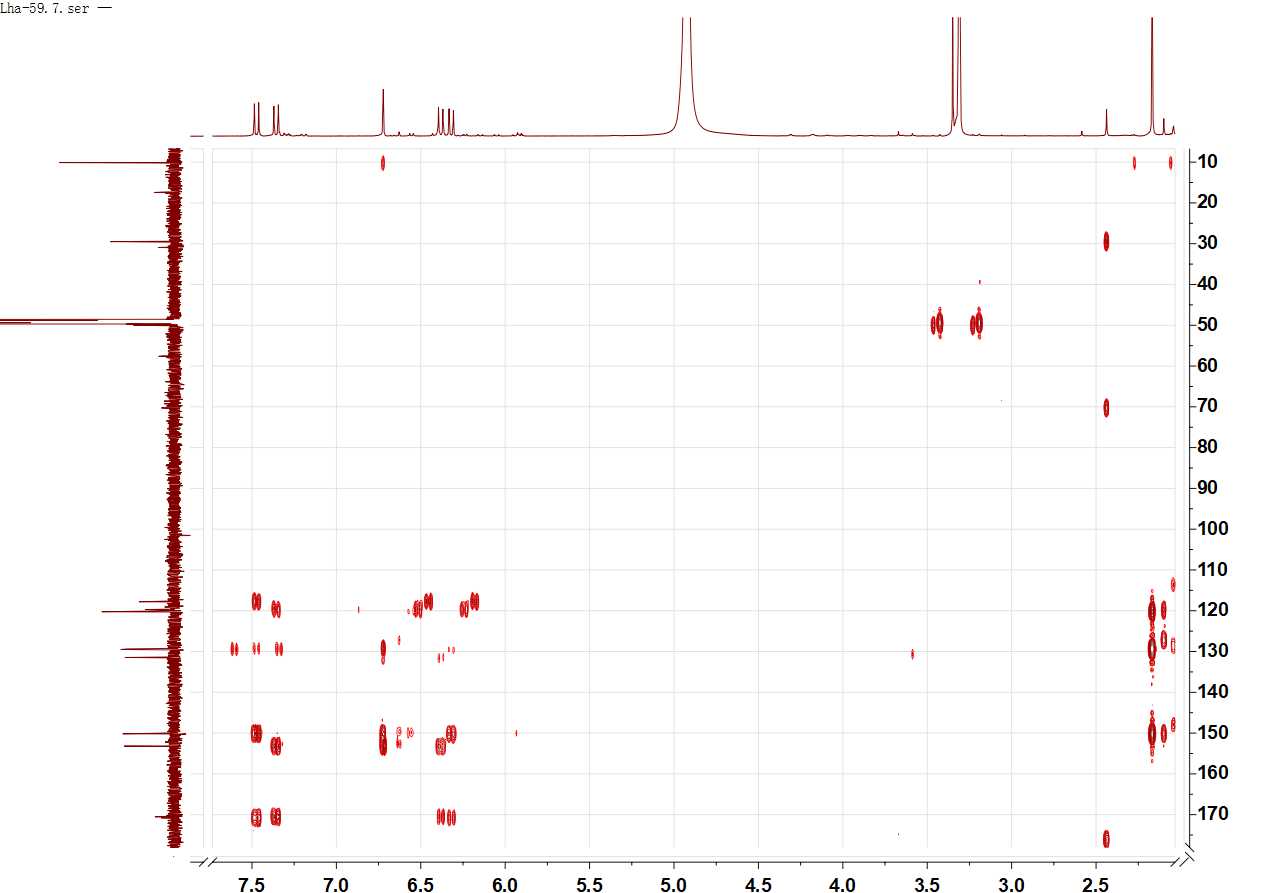


# Figure S12. HREIMS report of 2.


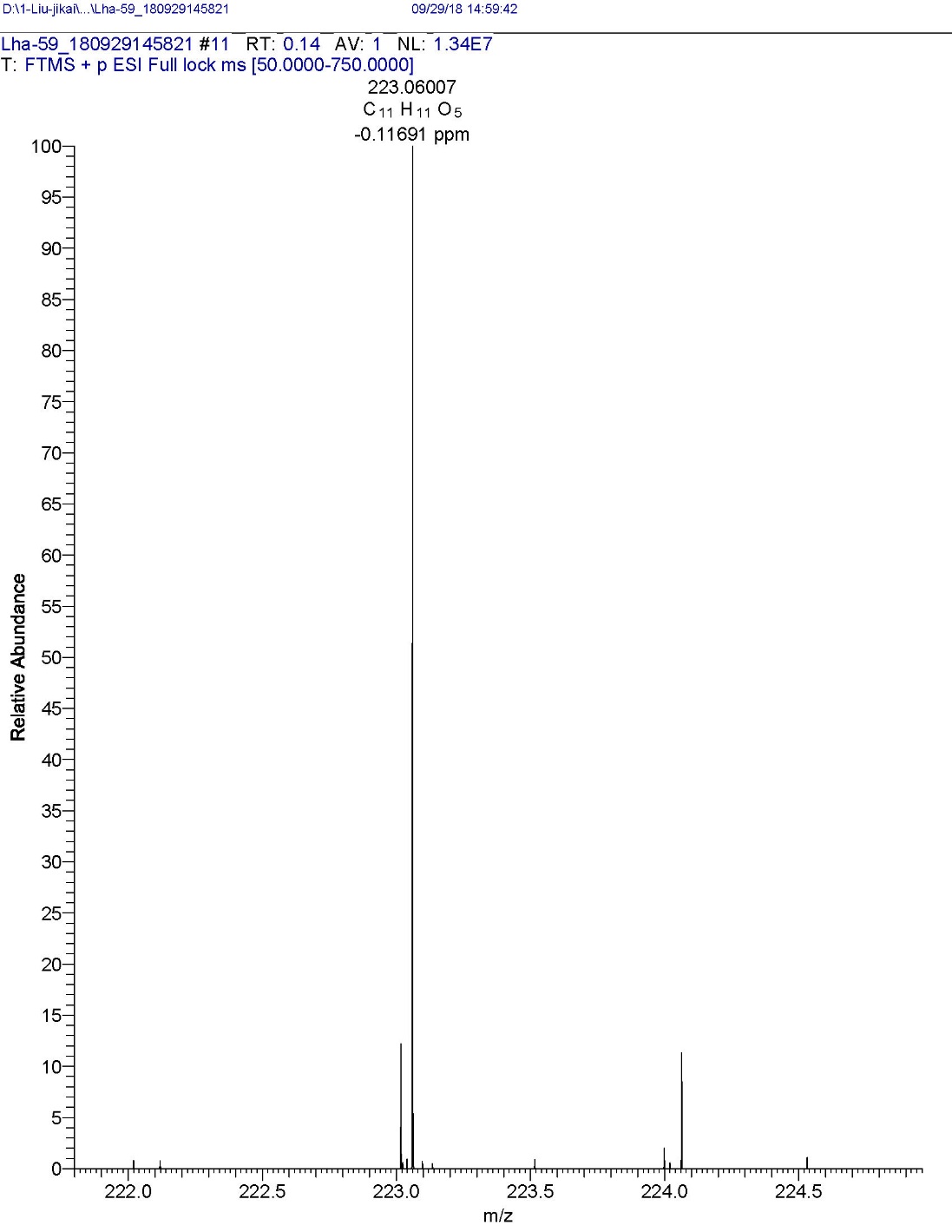


# Figure S13. ^1^H NMR spectrum of 3 (600 MHz, CD_3_OD).


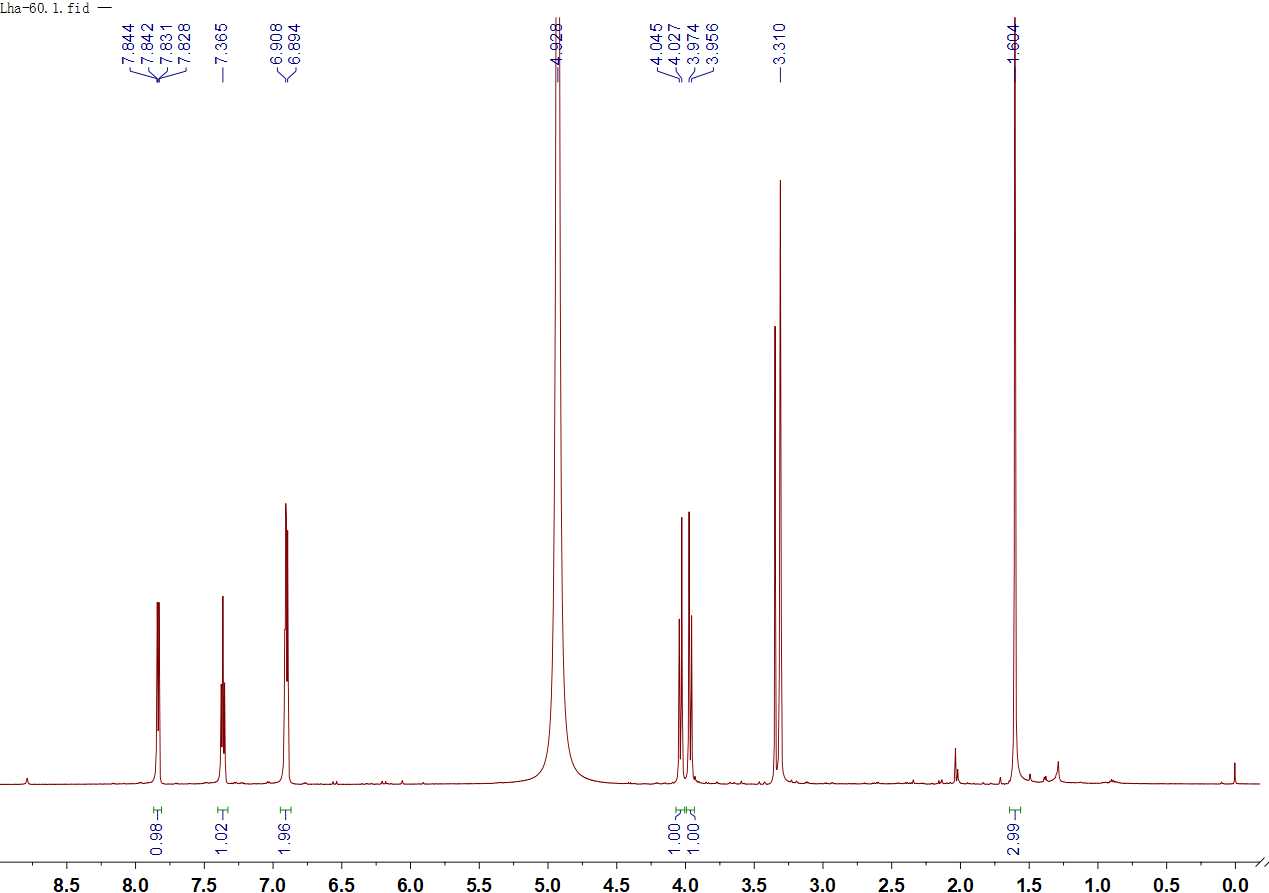


# Figure S14. ^13^C NMR and DEPT spectra of 3 (150 MHz, CD_3_OD).


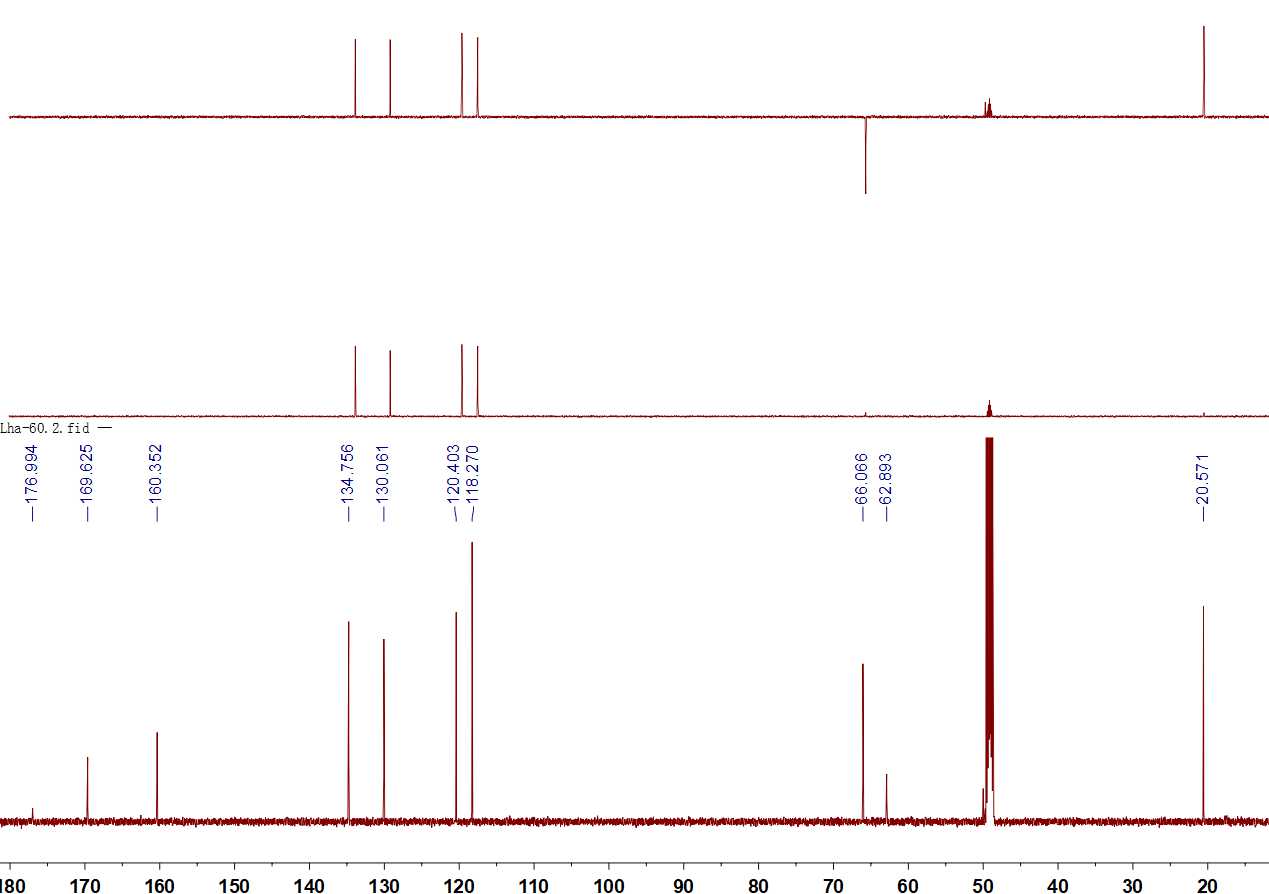


# Figure S15. HSQC spectrum of 3.


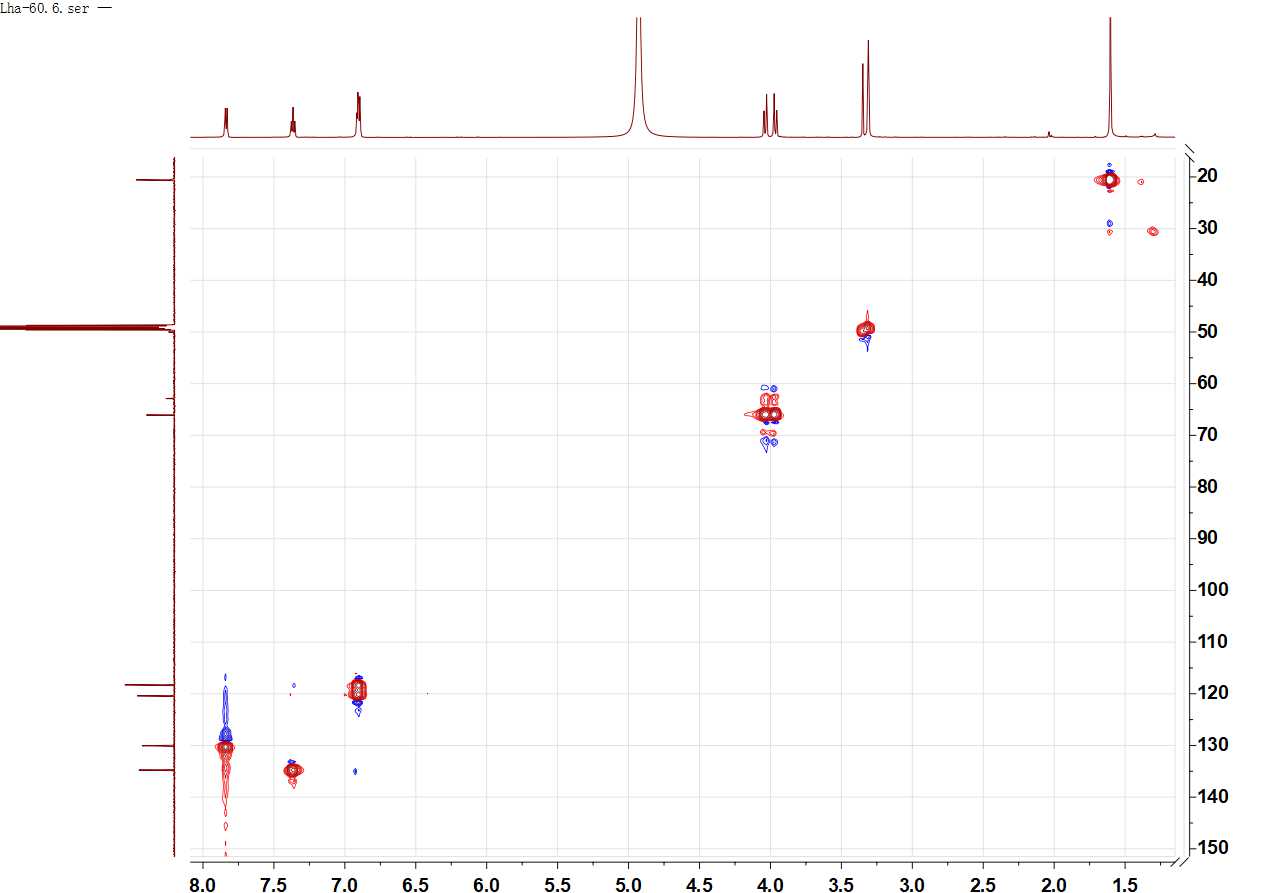


# Figure S16. ^1^H-^1^H COSY spectrum of 3.


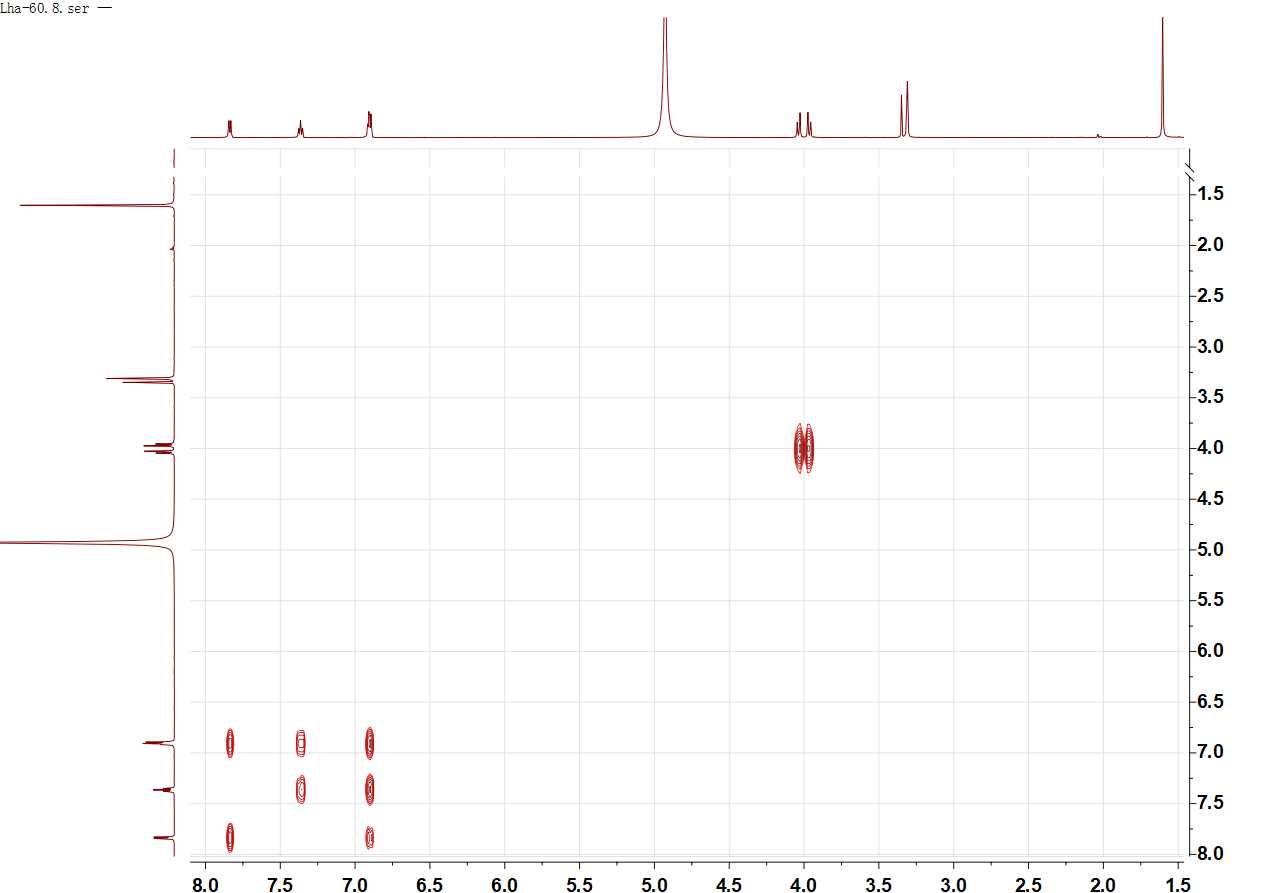


# Figure S17. HMBC spectrum of 3.


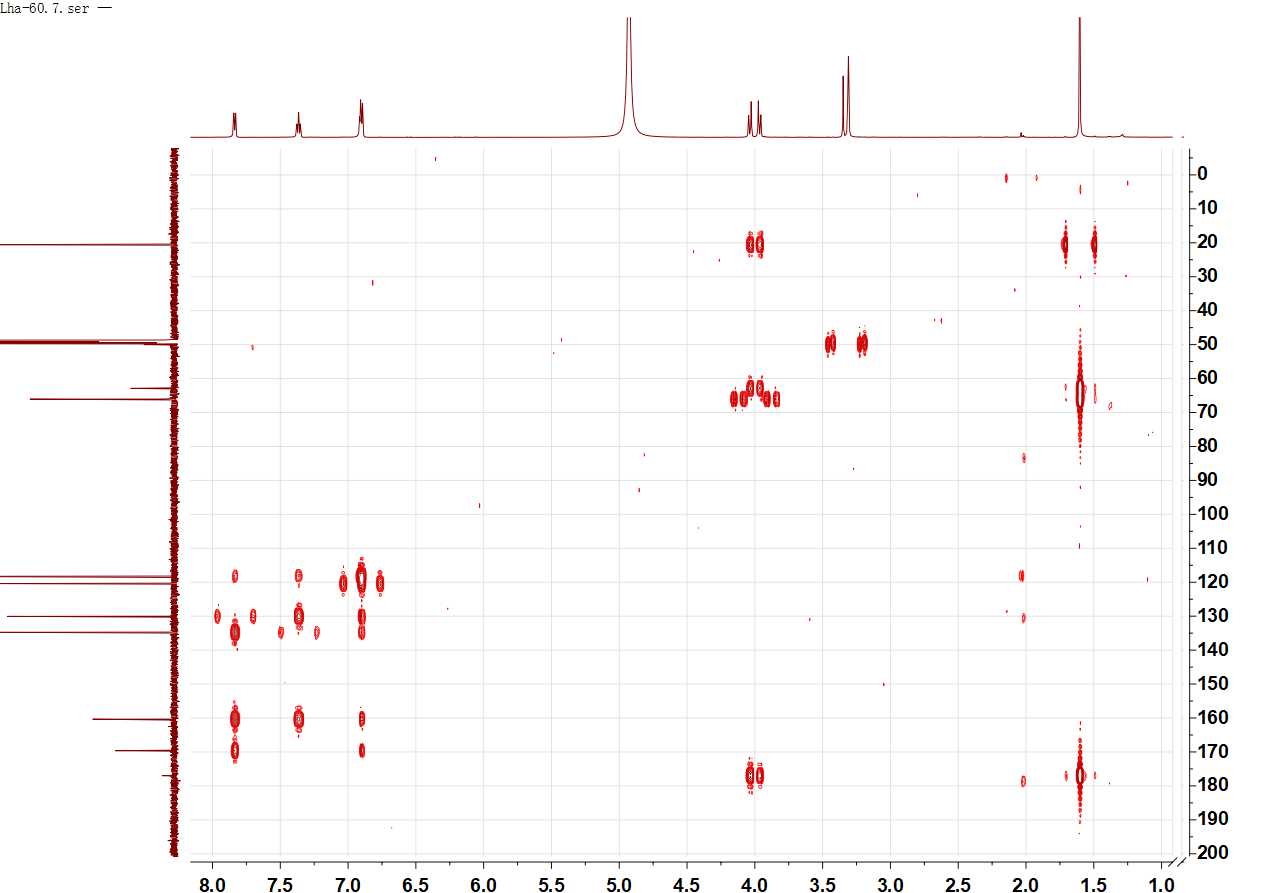


# Figure S18. HREIMS report of 3.


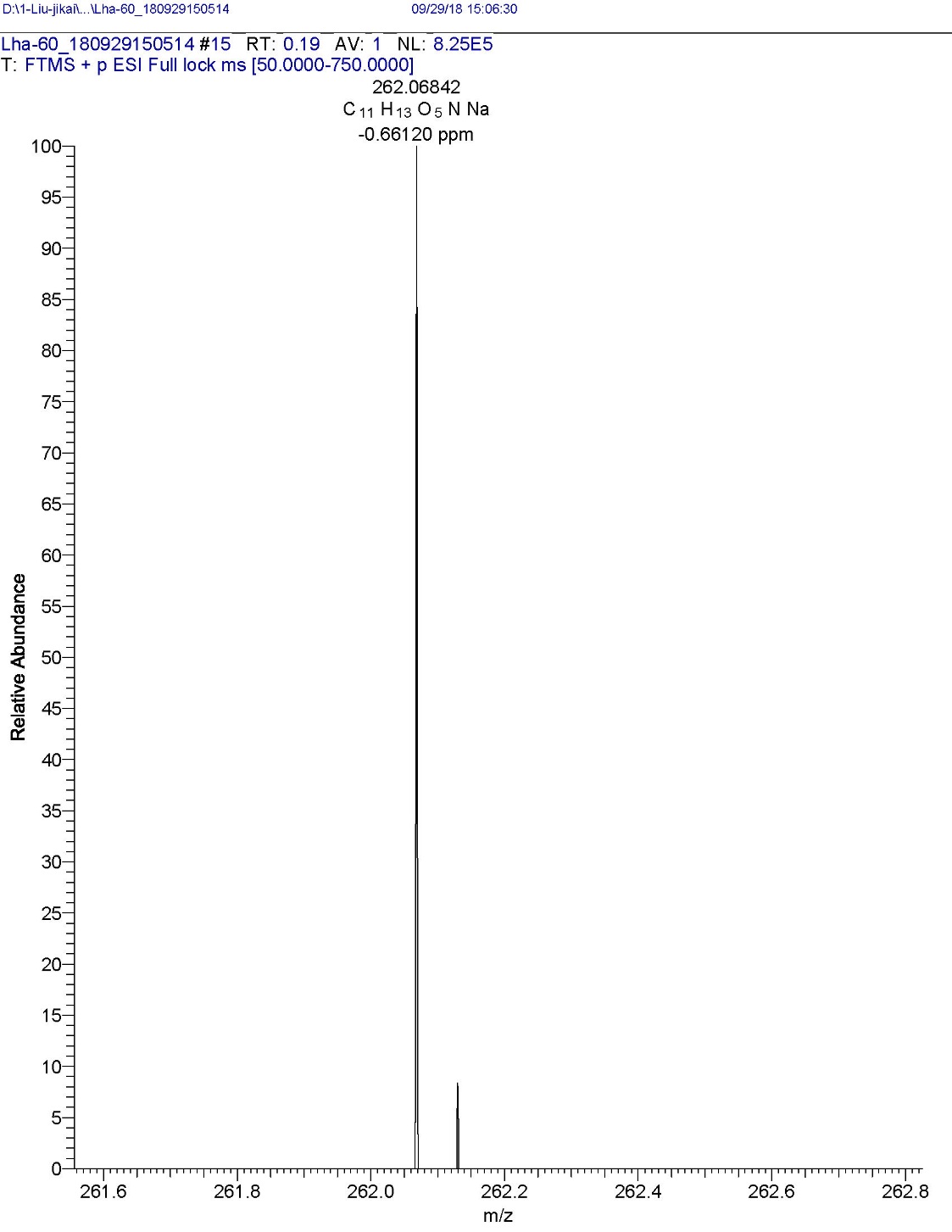


# Figure S19. Chiral-phase HPLC analysis of 3.

*n*-Hexane:isopropanol = 90:10, 1 mL/min.
